# Genome-Wide Informative Microsatellite Markers and Population Structure of *Fusarium virguliforme* from Argentina and the USA

**DOI:** 10.3390/jof9111109

**Published:** 2023-11-16

**Authors:** Leandro Lopes da Silva, Huan Tian, Brandi Schemerhorn, Jin-Rong Xu, Guohong Cai

**Affiliations:** 1 Crop Production and Pest Control Research Unit, Agricultural Research Service, United States Department of Agriculture (USDA), West Lafayette, IN 47907, USA; 2 Botany and Plant Pathology Department, Purdue University, West Lafayette, IN 47907, USA

**Keywords:** soybean, sudden death syndrome, *Fusarium virguliforme*, microsatellite marker, population structure, Argentina, USA

## Abstract

Soybean sudden death syndrome (SDS) is a destructive disease that causes substantial yield losses in South and North America. Whereas four *Fusarium* species were identified as the causal agents, *F. virguliforme* is the primary SDS-causing pathogen in North America and it also contributes substantially to SDS in Argentina. In this study, we comparatively analyzed genome assemblies of four *F. virguliforme* strains and identified 29 informative microsatellite markers. Sixteen of the 29 markers were used to investigate the genetic diversity and population structure of this pathogen in a collection of 90 strains from Argentina and the USA. A total of 37 multilocus genotypes (MLGs) were identified, including 10 MLGs in Argentina and 26 in the USA. Only MLG2, the most dominant MLG, was found in both countries. Analyses with three different approaches showed that these MLGs could be grouped into three clusters. Cluster IA consisting of four MLGs exclusively from the USA has much higher genetic diversity than the other two clusters, suggesting that it may be the ancestral cluster although additional data are necessary to support this hypothesis. Clusters IB and II consisted of 13 and 21 MLGs, respectively. MLGs belonging to these two clusters were present in all four sampled states in Argentina and all five sampled states in the USA.

## 1. Introduction

Sudden death syndrome (SDS) is a serious, growing threat to soybean production worldwide. In several countries including Argentina, Brazil, and the USA, this disease is one of the most limiting factor for soybean production [1,2]. In the last 10 years (2013 to 2022), SDS was estimated to have caused $3.35 billion yield loss in the USA, primarily in the northcentral states (Crop Protection Network, https://loss.cropprotectionnetwork.org (accessed on 22 September 2023). In Argentina, SDS can cause losses between 20 and 40%, reaching up to 90% of the soybean production [1].

Four species in the fungal genus *Fusarium*, *F. virguliforme*, *F. tucumaniae*, *F. brasiliense* and *F. crassistipitatum*, have been identified as the causal agents of SDS. In North America, SDS is primarily caused by *F. virguliforme* though *F. brasiliense* was recently reported in Michigan [3]. In South America, all four pathogens have been reported to cause SDS [4]. *F. tucumaniae* was found more often in South America than other species but a substantial portion of SDS-causing *Fusarium* strains from Argentina were identified as *F. virguliforme* [5,6]. *F. virguliforme* and *F*. *brasiliense* were reported to cause SDS in South Africa [7,8]. *F. virguliforme* was found in the soil in Malaysia [9], but the disease itself was not reported.

*F. virguliforme* is a soil-borne pathogen. It infects soybean roots and causes root rot. It can reach the lower stem but has never been found in the upper canopy. The pathogen produces phytotoxins [10,11,12] that cause leaf symptoms, including interveinal chlorosis, and premature defoliation with leaf petioles remaining intact on the stem.

To date, there are only limited and contradictory studies on the genetic diversity and population structures in *F. virguliforme*. Malvick and Bussey sequenced the internal transcribed space region and intergenic spacer region of nuclear rDNA as well as the translation elongation factor 1α (EF-1α) gene of *F. virguliforme* isolates from Minnesota, Illinois, Iowa and Missouri. They found little genetic diversity and concluded that these isolates were likely part of the same clonal population [13]. In another study, *F. virguliforme* isolates from Illinois, Iowa and Minnesota were divided into four subgroups based on analyses of four different molecular markers (Mitochondrial RFLP, mini- and micro-satellite PCR, (CAT)_5_ and (CAC)_5_ probes, and RAPD) [14]. Microsatellite markers were used to analyze 42 *F. virguliforme* isolates, mostly from Michigan [15]. Thirty-one multilocus genotypes (MLGs) were identified based on 12 microsatellite loci and these MLGs were grouped into three clusters that were not related to sampling sites.

Microsatellites, also called simple sequence repeats (SSR) or short tandem repeats (STRs), refer to tracts of repetitive, short (usually 1–6 bp) motifs in an organism’s genome. These regions are abundant and have higher rate of mutation [16], making microsatellites a robust and versatile tool in many biological fields, such as molecular-assisted breeding, population genetics, genealogy and genome mapping. Traditionally, there were two time- and resource-consuming bottlenecks in the development of microsatellite markers. The first bottleneck was to obtain microsatellite-containing sequences, which now can be carried out by mining raw or assembled genome sequences (e.g., [15,17]). The second bottleneck was that investigators had to experimentally screen a large number of microsatellite loci to obtain a small number of informative loci (the loci that showed genetic diversity in a species or population). With genome sequences of multiple individuals/isolates become available for many species, a comparative genomics approach has been developed to identify informative microsatellite markers without the need of experimental screening [17].

In this study, we aimed to use comparative genomics approach to identify informative microsatellite markers in *F. virguliforme* and use these markers to investigate genetic diversity and population structure of this pathogen in the United States and Argentina, two countries in which SDS has been causing substantial yield loss for soybean growers.

## 2. Materials and Methods

### 2.1. Bioinformatics Analysis

Informative microsatellite markers were identified following the previously published protocol [17]. Genome assemblies of four strains of *F. virguliforme* were downloaded from GenBank: Mont-1 (GenBank WGS No. AEYB01), NRRL34551 (MADZ01), LL0009 (MADY01) and Clinton-1B (MADX01). Microsatellite loci were identified in the genome of strain Mont-1 using the software package MISA version 2.1 [18] with the following parameters: for mononucleotide motifs, minimal 8 repeats; dinucleotide motifs, minimal 6 repeats; tri- to hexa- nucleotide motifs, minimal 5 repeats; and minimal distance between neighboring loci for identification of compound loci, 0 bp. Up to five primer pairs were designed for all loci with di- to hexa- nucleotide motifs using Primer3 version 2.5.0 [19] with the following requirements: product size 100–250 bp, primer length 18–25 bp (optimal 20 bp), primer melting temperature 57–63 °C (optimal 60 °C), primer GC ratio 30–70% (optimal 50%). All primers were required to be located within 200 bp in the flanking regions but with a minimal 3 bp away from the target locus. If there was another microsatellite locus within the 200 bp flanking regions of a target locus, the primers should not overlap with the neighboring locus.

In silico PCR was conducted using the software package isPcr version 3.3 [20] with default parameters in all four genome assemblies (Mont-1, LL0009, NRRL34551, and Clinton-1B) with the designed primer pairs. A custom PERL script was then used to process the in silico PCR results and select loci that fitted the following criteria: (1) only one PCR amplicon was generated from each strain; (2) the amplicons matched the primer sequences perfectly on each end; (3) the amplicons contained the same microsatellite motif but differ in number of repeats; (4) for loci with 2–3 bp motifs, the length of flanking regions were identical in all four strains; and (5) for loci with 4–6 bp motifs, 1 bp difference was allowed in the length of the flanking regions.

### 2.2. Fungal Strains, DNA Manipulation and Microsatellite Genotyping

A total of 90 strains of *F. virguliforme* were used in this study (Table 1). The 56 strains from the USA were obtained from Agricultural Research Service Culture Collection (NRRL–Northern Regional Research Laboratory, https://nrrl.ncaur.usda.gov/ (accessed on 20 October 2019)) as well as our own collection. The 34 Argentina isolates were provided by Dr. Mercedes Scandiani from Rosario National University, Argentina. The strains were cryopreserved in glycerol in liquid nitrogen and maintained on Potato Dextrose Agar (PDA). For DNA extraction, mycelium was harvested from seven-day-old cultures in potato dextrose broth grown at room temperature on benchtop. DNA was extracted from harvested mycelium using the Plant Dneasy Mini kit (Qiagen, Germantown, MD, USA) following the manufacturer’s instructions. A species-specific conventional PCR was conducted for all strains to confirm *F. virguliforme* identity following previously published protocol [21], with an initial denaturing cycle at 95 °C for 5 min, followed by 35 cycles of denaturing at 95 °C for 30 s, annealing at 65 °C for 30 s, extension at 72 °C for 30 s, and a final extension cycle at 72 °C for 5 min.

The primers for the microsatellite markers were first validated on the four strains whose genome sequences were used in the bioinformatic data mining described above (Mont-1, NRRL34551, LL0009, and Clinton-1B) before they were used to genotype all strains. For microsatellite genotyping, PCR reactions were performed with three primers: forward primer with a M13 sequence (TGTAAAACGACGGCCAGT) appended to its 5′ ends, reverse primer, and M13 primer labeled with 5′-FAM. PCR reactions were performed in duplicates in 15 μL containing 1× Amplitaq Gold Buffer (Applied Biosystems, Waltham, MA, USA), 2 mM MgCl_2_, 0.2 mM mixed dNTPs, 0.6 U Amplitaq Gold DNA Polymerase (Applied Biosystems, Waltham, MA, USA), 40 nM forward primer, 160 nM 5′FAM-labeled M13 primer and 160 nM reverse primer, and 10 ng template DNA. An initial denaturing cycle at 95 °C for 5 min was followed by 38 cycles of denaturing at 95 °C for 30 s, annealing for 45 s at 58 °C for the first 25 cycles and 54 °C for the remaining 13 cycles, and extension at 72 °C for 45 s, and a final extension cycle at 72 °C for 15 min. Two PCR reactions were performed for each strain. The sizes of PCR amplicons were resolved using an ABI 3730XL Genetic Analyzer together with GeneScan™ 600 LIZ™ dye Size Standard v2.0 (Applied Biosystems, Waltham, MA, USA). Allele sizes were determined with the aid of the software Peak Scanner version 2.0 (Applied Biosystems, Waltham, MA, USA). To determine the number of repeats, amplicon sizes in individual strains were compared to the amplicon size in Mont-1.

### 2.3. Data Analysis

Strains were assigned to MLGs based on the number of repeats in 16 microsatellite loci. Population structure was assessed by Bayesian clustering in Structure version 2.3.4 [22]. The parameters were an admixture model, at 100,000 burn-in followed by 100,000 Markov Chain Monte Carlo simulations with 10 replications for each K (ranging from K = 1 to K = 10). The online tool Structure Harvester (https://taylor0.biology.ucla.edu/structureHarvester/ (accessed on 23 June 2023)) was used to select the optimal K from the results obtained from Structure [23,24]. A dendrogram was constructed using a unweighted pair group method with arithmetic mean (UPGMA) method with Euclidian distance and 10,000 bootstrap in Past software version 4.03 [25]. A genetic distance was calculated for all MLG and used for principal coordinate analysis (PCoA) in GENALEX version 6.502 [26]. A genotype accumulation curve was constructed using the poppr package in R software version 4.1.2 [27]. This R package was also used to calculate Nei’s gene diversity [28]. Genetic variation between populations was tested with Analysis of MOlecular VAriance (AMOVA) implemented in Arlequin version 3.5.2.2 [29]. The network connection output data generated from Arlequin [29] was used in HapStar version 0.5 to create a minimum spanning tree [30].

## 3. Results

### 3.1. Microsatellite Loci in F. virguliforme

A total of 9620 microsatellite loci were identified in the genome assembly of Mont-1 (Table S1). The microsatellite density is 190.7 loci per Mb genome. Sixty-four loci are in compound formation (two or more loci immediately next to each other). The number of loci with mono- to hexa- nucleotide motifs are 5470, 1722, 2024, 307, 45, and 52, respectively. The most common mono- to hexa-nucleotide motifs are A/T (3759), AT/AT (809), AAT/ATT (774), AATT/AATT (148), ACGAG/CGTCT and ATCCC/ATGGG (4 each), and AACAGC/CTGTTG (4), respectively.

### 3.2. Identification of Informative Microsatellite Loci in F. virguliforme

Of the 4078 microsatellite loci with di- to hexa- motifs that are not in compound form, primer pairs were successfully designed for 2671 loci. Primer3 was able to design five primer pairs for 2664 loci, but only one and two primer pairs for one and six loci, respectively. No primer pairs could be designed for the remaining 1407 loci due to the lack of sufficient flanking sequences (too close to contig ends or to neighboring microsatellite loci), and/or low complexity of their flanking sequences. 

Processing of in silico PCR results identified 2625 loci with one amplicon per genome assembly (including missing amplicon in one or more assemblies) but with identical repeat numbers, and 38 loci with different repeat numbers. Among those 38 loci, 9 loci were predicted to produce amplicons in which the flanking sequences had length differences exceeding the threshold among the four *F. virguliforme* strains. Hence, the comparative genomics approach identified 29 informative loci that met all of the requirements laid out in Section 2.1 “Bioinformatics analysis” and 133 primer pairs for these loci (Table 2). One primer pair was randomly chosen for each locus for population study below (Table S2). Eleven of the 29 loci had missing predicted amplicon in one or two strains based on in silico PCR due to fragmentation of genome assemblies. PCR successfully amplified an amplicon of expected size in all four strains (Figure S1).

### 3.3. Population Structure

Species-specific PCR confirmed all strains as *F. virguliforme* with an amplicon of expected size (Figure S2 and not shown). For the 16 microsatellite markers used to investigate genetic diversity and population structure in these 90 strains, an allele was successfully amplified for each locus in each strain. Two to ten alleles were detected for each locus. Based on the number of repeats in the 16 loci, the 90 strains were assigned to 37 MLGs (Table 3). MLG2 was most dominant represented by 25 strains (15 from Argentina and 10 from the USA), followed by MLG3 (8 strains, all from Argentina) and MLG14 (8 strains, all from the USA). One to five strains were found in other MLGs including 26 MLGs represented by only a single strain. Of the 37 MLGs, 10 were only found in Argentina and 26 were only found in the USA. Other than MLG2, no other MLG was found in both countries (Table 1). Genotype accumulation curve showed that 15 loci were able to detect all 37 MLGs (Figure S3).

**Figure 1:**
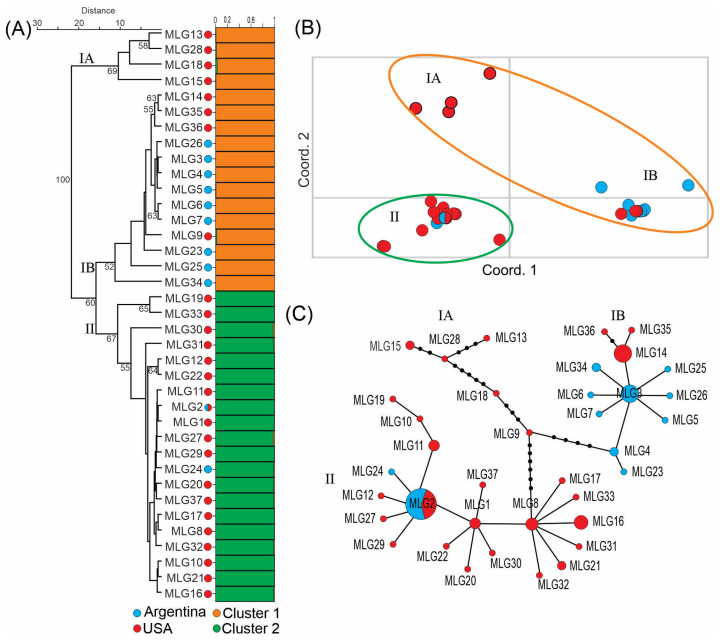
Population structure of *Fusarium virguliforme* strains from Argentina and the USA. (**A**) Right side, Bayesian cluster analysis assigned the 37 MLGs into 2 clusters. The abundance of orange and green color on the horizontal bars indicates the likelihood of individual MLGs belonging to a particular cluster. Left side, UPGMA dendrogram grouped the 37 MLGs into 3 clusters. Numbers indicate bootstrap support in percentile. (**B**) Principal coordinate analysis (PCoA). (**C**) Minimum spanning tree. The areas of the circles are proportional to the number of strains in each MLG. Small black dots on the lines represent additional differences between neighboring MLGs.

Bayesian analysis predicted that the 37 MLGs most likely belonged to two clusters (Figure S4). Grouping of the 37 MLGs into clusters I and II was shown in Figure 1A (right side). However, UPGMA dendrogram grouped the 37 MLGs into three clusters, IA, IB and II, with over 60% bootstrap support (Figure 1A, left side). The latter was supported by PCoA analysis (Figure 1B) and the minimal spanning tree (Figure 1C). Four MLGs, 13, 15, 18 and 28, all from the USA, formed cluster IA. Cluster IB consisted of nine MLGs (18 strains) from Argentina and four MLGs (11 strains) from the USA. Cluster II consisted of 19 MLGs (40 strains) from the USA and two MLGs (16 strains) from Argentina, including MLG2 that was found in both countries (Figure 1 and Table 1).

The Argentina population was significantly different from the population from the USA based on AMOVA analysis. The differences between these two populations contributed to 17.20% of variance when analyzed by strains and 37.11% of variance when analyzed by MLGs (clone-correction), which is statistically significant (*p* < 0.05) for both analyses (Table 4). These two populations had similar genetic diversity based on Nei’s gene diversity index, with 0.31 for Argentina population and 0.33 for the population from the USA. Cluster IA had much higher diversity (0.30) than cluster IB (0.11) and cluster II (0.10) (Table 5).

## 4. Discussion

Advances in genomics removed the need of a tedious, and time- and resource-consuming bottleneck in the traditional method of developing microsatellite markers, namely obtaining microsatellite-containing sequences through construction and sequencing of microsatellite-enriched libraries [31,32,33]. However, investigators still faced another bottleneck, a large number of markers had to be experimentally screened to obtain a small number of informative markers. For example, in *Anisogramma anomala*, a fungus that causes eastern filbert blight on hazelnut trees, 236 loci were screened and 23 loci were found to be informative and suitable for future studies. In *F. virguliforme*, 12 informative loci were identified by screening of 92 loci [15]. Nine of the twelve loci were also identified in this study (Table S3).

Taken advantage of the availability of multiple genomes in many species, we previously developed a comparative genomics approach to identify informative microsatellite loci by bioinformatically mining genomes of multiple individuals in a species and applied it to *Phytophthora sojae*, an oomycete that causes Phytophthora root rot in soybean [17]. In silico PCR, a useful tool that allowed us to simulate experimental PCR based on genome assemblies, was essential for this approach. By comparing genome assemblies of 4 strains, a total of 157 informative microsatellite loci were identified in *P. sojae*. Experimental screening of 20 loci validated the results from data mining. In this study, we applied the same approach to *F. virguliforme* and identified 29 informative loci. Population study using 16 of the resulting loci supported the finding that the comparative genomics approach resolved the second bottleneck by eliminating the need to experimentally screening a large number of loci.

The quality of genome assemblies has a big impact on the informative microsatellite loci identification by the comparative genomics approach. The four assemblies used in this study had similar sizes (49.4–50.9 Mb) but they were all very fragmented. Whereas the assembly of strain Mont-1 consisted of 3098 contigs, the assemblies of the other three strains each consisted of over 20 thousand contigs. Mont-1 was sequenced using Roche 454 technology and assembled using PCAP.Rep.454 software version May-2010 and the other three strains were all sequenced using Illumina HiSeq technology and assembled using PCAP.Solexa software version Jan-2014. This fragmentation was the primary reason for the failure to design suitable primers for 1407 of 4078 loci. We found that 2625 of the 2671 loci had identical repeat numbers but many loci did not have a predicted amplicon in in silico PCR analysis with one or more strains, also mainly due to the fragmentation of the genome assemblies. Eleven of the 29 informative loci identified in this study had no predicted amplicon in one or two strains based on in silico PCR analysis but experimental PCR successfully amplified an allele in each strain. If the genome assemblies had been more continuous, we expect that primers could be designed for more loci. If we had predicted amplicon in all four strains for the 2625 which did not show polymorphism in in silico PCR, some loci might turn out to be informative.

Another factor that impacts the result of comparative genomics approach is the genetic diversity of the individuals used in the analysis. In our analysis, we had strains from both Argentina (NRRL34551) and the USA (Mont-1, LL0009 and Clinton-1B). They turned out to be in all three clusters, NRRL34551 (MLG34, cluster IB), Mont-1 (MLG14, cluster IB), LL0009 (MLG20, cluster II), and Clinton-1B (MLG15, cluster IA). If any one strain was excluded from the analysis, some loci would not be identified as informative (Table 2). We also detected more alleles for some loci in the population study than bioinformatic prediction (Table 2 and Table 3). For example, 8 and 10 alleles were detected for loci SSR20 and SSR23, respectively, in the population study, while only two and three alleles, respectively, were predicted by bioinformatic analysis. With more and better genomes being included in the bioinformatic analysis, more informative microsatellite loci will likely be identified.

We used 16 microsatellite loci to investigate a large collection of strains from Argentina and the USA. UPGMA dendrogram, PCoA analysis and minimal spanning tree all supported the grouping of these strains into three clusters. The four MLGs in cluster IA, all from the USA, had much higher genetic diversity than the other two clusters (Table 5). These MLGs differed from the two major clusters (IB and II) in multiple loci and differed from each other in multiple loci (Figure 1C and Table 3). Based on UPGMA dendrogram, cluster IA was basal to cluster IB and II (Figure 1A, left side). These data suggested that cluster IA represented the ancestral genetic diversity in *F. virguliforme* from where two major lineages, cluster IB and cluster II, evolved. O’Donnell et al. theorized that SDS-causing *Fusarium* pathogens originated from South or Mesoamerica based on the fact that soybean was introduced into South America earlier than North America and that more SDS-causing *Fusarium* species were found in South America than in North America [6]. Our results do not support this contention in the case of *F. virguliforme*. More studies are required to resolve this issue.

Cluster IB consisted primarily of MLGs from Argentina. The Argentina MLGs in this cluster seemed to be evolved around MLG3. Other Argentina MLGs differed from MLG3 by one or two loci (Figure 1C and Table 3). It’s interesting to note that MLG3 and MLG14, with eight strains each (second only to MLG2), were in cluster IB and differed from each other in only one locus (SSR17), but they were found exclusively in Argentina and the USA, respectively.

Cluster 2 consisted primarily of MLGs from the USA. MLG2, the most dominant MLG and the only MLG that was found in both countries, belong to this cluster. The MLGs from the USA in this cluster were diverse but the two MLGs from Argentina in this cluster differed from each other by only one locus (MLG2 and MLG24, SSR23). The facts that clusters IB and II mainly consist of MLGs from Argentina and the USA, respectively, and only one MLG is common in both countries, suggested that there is only limited genetic exchange between *F. virguliforme* isolates from South and North America due to geographic isolation. An alternative explanation is that environmental factors in Argentina may favor cluster IB while cluster II is more adapted to the environments of the USA. However, at the strain level, more strains in cluster II (18 strains) were found in Argentina than strains in cluster IB (16 strains).

**Figure 2:**
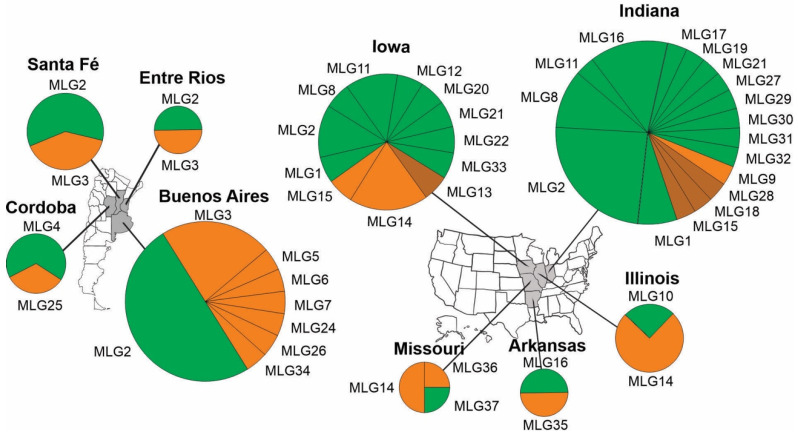
Geographic distribution of multilocus genotypes (MLGs) in individual states in Argentina and the USA. Three strains, two from Argentina and one from the USA, whose state origin unknown (Table 1) were not included in the analysis. Brown, MLGs in cluster IA; orange, MLGs in cluster IB; and green, MLGs in cluster II (Figure 1). The areas of the circles are proportional to the number of strains from individual states and the areas of the pies are proportional to the number of strains in individual MLGs from individual states.

At the state level, MLGs in cluster IB and cluster II were found in all four states in Argentina and all five states in the USA. MLG2, the most dominant MLG in both countries, were found in three states in Argentina (Santa Fe, Entre Rios and Buenos Aires) and two states in the USA (Indiana and Iowa). In Argentina, about half of the strains originated from those three states belonged to MLG2. Indiana was the most sampled state in both countries, with 29 strains belonging to 17 MLGs. Strains and MLGs in cluster II dominated the Indiana collection, and to a lesser extent, the Iowa collection. MLGs in cluster IA were found only in Indiana and Iowa (Figure 2).

To our knowledge, this is the first study that examined the population structure of *F. virguliforme* in a large and broad collection of strains from both Argentina and the USA. It showed that our collection likely consisted of three clusters, a potentially ancestral cluster that was only found in the USA, and two dominant clusters that were found in all sampled states in both countries. More studies with broader collection from North and South America are needed to determine the origin of this pathogen. Host resistance is the most economic and effective means in managing soil-borne diseases such as SDS [34]. For breeding programs, it is important to evaluate the soybean lines under development using representative isolates of the pathogen. Our study suggests that the dominant MLGs in Cluster IB (MLG3 and MLG14) and cluster II (MLG2) and a representative strain in cluster IA would be a good choice.

## Figures and Tables

**Table 1:** *Fusarium virguliforme* strains used in this study.

Strain	Country	State	City or County *	Multilocus Genotype
11-385-16	Argentina	Buenos Aires	Fontezuela	MLG2
11-389-1	Argentina	Buenos Aires	Fontezuela	MLG2
11-389-3	Argentina	Buenos Aires	Fontezuela	MLG2
11-389-5	Argentina	Buenos Aires	Fontezuela	MLG2
11-389-7	Argentina	Buenos Aires	Fontezuela	MLG2
11-389-9	Argentina	Buenos Aires	Fontezuela	MLG2
11-390-1	Argentina	Buenos Aires	Fontezuela	MLG2
11-390-10	Argentina	Buenos Aires	Fontezuela	MLG3
11-390-4	Argentina	Buenos Aires	Fontezuela	MLG24
11-390-8	Argentina	Buenos Aires	Fontezuela	MLG2
11-391-3	Argentina	Buenos Aires	Fontezuela	MLG2
11-392-1	Argentina	Buenos Aires	Fontezuela	MLG2
11-392-2	Argentina	Buenos Aires	Fontezuela	MLG2
11-420-3	Argentina	Buenos Aires	Ines Indart	MLG3
11-421-5	Argentina	Buenos Aires	Ines Indart	MLG3
NRRL36610	Argentina	Buenos Aires	Pergamino	MLG3
NRRL36611	Argentina	Buenos Aires	Pergamino	MLG3
NRRL34551	Argentina	Buenos Aires	San Pedro	MLG34
12-274p1	Argentina	Buenos Aires	San Pedro	MLG6
12-274p2	Argentina	Buenos Aires	San Pedro	MLG7
12-274-p4	Argentina	Buenos Aires	San Pedro	MLG26
12-257	Argentina	Buenos Aires	Del Socorro	MLG5
11-512-4	Argentina	Cordoba	Gral. Roca	MLG25
11-408-2	Argentina	Cordoba	Leones	MLG4
11-408-4	Argentina	Cordoba	Leones	MLG4
11-393-3	Argentina	Entre Rios	Diamante	MLG2
11-393-N	Argentina	Entre Rios	Diamante	MLG3
11-385-12	Argentina	Santa Fe	Armstrong	MLG2
11-385-13	Argentina	Santa Fe	Armstrong	MLG2
11-385-15	Argentina	Santa Fe	Armstrong	MLG2
11-385-5	Argentina	Santa Fe	Armstrong	MLG3
NRRL36897	Argentina	Santa Fe	Los Molinos	MLG3
NRRL54529	Argentina	-	-	MLG34
11-385-2-1	Argentina	-	-	MLG23
NRRL37585	USA	Arkansas	-	MLG16
NRRL37586	USA	Arkansas	-	MLG35
NRRL31039	USA	Illinois	Champaign	MLG10
NRRL22292	USA	Illinois	-	MLG14
LL0094	USA	Illinois	-	MLG14
Mont-1	USA	Illinois	-	MLG14
12IN-ADAMS	USA	Indiana	Adams	MLG8
14-INS-27	USA	Indiana	Boone	MLG11
14-INS-30	USA	Indiana	Carroll	MLG2
Clinton1-b	USA	Indiana	Clinton	MLG15
14-INS-16-1	USA	Indiana	Fayette	MLG2
14-INS-26	USA	Indiana	Jennings	MLG8
14-INS-25	USA	Indiana	LaPorte	MLG30
14-INS-29	USA	Indiana	Miami	MLG32
INMO-A1	USA	Indiana	Monon	MLG2
INMO-A7	USA	Indiana	Monon	MLG16
INMO-D4	USA	Indiana	Monon	MLG2
INMO-E1	USA	Indiana	Monon	MLG17
INMO-E5	USA	Indiana	Monon	MLG18
INMO-G1	USA	Indiana	Monon	MLG8
INMO-G4	USA	Indiana	Monon	MLG1
14-INS-19	USA	Indiana	Parke	MLG27
14-INS-24	USA	Indiana	Pulaski	MLG2
14-INS-18	USA	Indiana	Sulivan	MLG2
14-INS-21	USA	Indiana	Vigo	MLG28
14-INS-28	USA	Indiana	Whitley	MLG31
INMO-P6	USA	Indiana	-	MLG19
INS-12-10-1	USA	Indiana	-	MLG16
INS-12-10-3	USA	Indiana	-	MLG16
NRRL22823	USA	Indiana	-	MLG16
NRRL22825	USA	Indiana	-	MLG9
NRRL37592	USA	Indiana	-	MLG21
00-11-18-3	USA	Indiana	-	MLG1
14-INS-17-1	USA	Indiana	-	MLG2
14-INS-22	USA	Indiana	-	MLG29
LL0028	USA	Iowa	Boone	MLG21
LL0036	USA	Iowa	Buchanan	MLG14
NRRL32460	USA	Iowa	Cerro	MLG33
NRRL32464	USA	Iowa	Clinton	MLG11
NRRL32466	USA	Iowa	Clinton	MLG12
LL0059	USA	Iowa	Clinton	MLG11
NRRL32468	USA	Iowa	Greene	MLG13
NRRL32470	USA	Iowa	Henry	MLG8
NRRL32471	USA	Iowa	Jasper	MLG2
NRRL32472	USA	Iowa	Jasper	MLG2
NRRL32479	USA	Iowa	Scott	MLG15
LL0009	USA	Iowa	Story	MLG20
LL0019	USA	Iowa	Washington	MLG14
NRRL32481	USA	Iowa	Worth	MLG1
LL0072	USA	Iowa	-	MLG22
LL0085	USA	Iowa	-	MLG14
NRRL32475	USA	Missouri	Mont	MLG14
NRRL32476	USA	Missouri	Mont	MLG14
NRRL37590	USA	Missouri	-	MLG36
NRRL37591	USA	Missouri	-	MLG37
HUMCH1	USA	-	-	MLG2

* Missing data were labeled as “-”.

**Table 2:** Informative microsatellite loci identified through bioinformatic analysis of genome assemblies of four *Fusarium virguliforme* strains.

Loci	Expected Amplicon Size in Mont-1	Number of Repeats ^a^				Motif
		Mont-1	NRRL34551	Clinton-1B	LL0009	
SSR1	187	8	16	14	-	(TTGCCA)
SSR2	176	8	5	4	-	(CCGTGG)
SSR3	150	13	12	12	12	(GT)
SSR4	227	13	33	35	19	(AAG)
SSR5	231	12	-	10	-	(ACG)
SSR6	258	22	12	21	22	(TG)
SSR7	197	13	13	14	13	(GAA)
SSR8	245	13	10	-	10	(CTGCTT)
SSR9	170	18	18	18	19	(TG)
SSR10	222	13	13	13	12	(TGT)
SSR11	128	21	20	21	18	(AC)
SSR12	141	10	10	9	10	(TTGC)
SSR13	220	18	18	18	9	(AAC)
SSR14	170	7	8	8	8	(CA)
SSR15	259	10	10	11	-	(TGTCTG)
SSR16	216	10	11	11	11	(GT)
SSR17	248	6	8	6	6	(AGCACA)
SSR18	263	8	8	8	4	(GAC)
SSR19	255	20	20	19	20	(TAT)
SSR20	192	15	15	-	23	(GTT)
SSR21	153	12	12	10	10	(CAGCAA)
SSR22	129	6	6	6	5	(CTT)
SSR23	250	30	30	20	35	(CAA)
SSR24	262	30	29	-	-	(TTG)
SSR25	179	11	11	-	6	(TCAC)
SSR26	222	22	20	-	20	(CTA)
SSR27	254	22	20	-	-	(TAT)
SSR28	262	6	6	11	6	(AT)
SSR29	157	6	6	-	5	(AT)

^a^ Missing predicted amplicons were labeled as “-”.

**Table 3:** Multi-locus genotypes identified in *Fusarium virguliforme* using 16 microsatellite loci.

ID	Number of Strains	Country	Number of Repeats															
			SSR5	SSR6	SSR7	SSR9	SSR10	SSR11	SSR12	SSR13	SSR15	SSR17	SSR20	SSR21	SSR22	SSR23	SSR25	SSR28
MLG1	3	USA	12	22	13	19	12	18	10	9	10	6	23	10	6	35	6	6
MLG2	25	ARG(15) USA(10)	12	22	13	19	12	18	10	9	10	6	23	10	6	36	6	6
MLG3	8	ARG	12	22	13	18	13	21	10	18	10	8	15	12	6	30	11	6
MLG4	2	ARG	12	22	13	18	13	21	10	18	10	8	15	12	6	31	11	6
MLG5	1	ARG	12	22	13	19	13	21	10	18	10	8	15	12	6	30	11	6
MLG6	1	ARG	12	22	13	18	15	21	10	18	10	8	15	12	6	30	11	6
MLG7	1	ARG	12	22	13	18	14	21	10	18	10	8	15	12	6	30	11	6
MLG8	4	USA	12	21	13	19	12	18	10	9	10	6	23	10	6	35	6	6
MLG9	1	USA	10	21	13	18	12	21	10	18	10	6	15	10	6	31	11	6
MLG10	1	USA	12	21	13	19	12	18	10	9	13	6	23	10	6	36	6	6
MLG11	3	USA	12	21	13	19	12	18	10	9	10	6	23	10	6	36	6	6
MLG12	1	USA	12	22	13	19	12	18	10	9	10	6	23	7	6	36	6	6
MLG13	1	USA	10	21	13	18	13	21	9	18	12	6	35	10	6	28	6	11
MLG14	8	USA	12	22	13	18	13	21	10	18	10	6	15	12	6	30	11	6
MLG15	2	USA	10	21	14	18	13	21	9	18	11	6	37	10	6	20	6	11
MLG16	5	USA	12	21	13	19	12	18	10	9	12	6	23	10	6	35	6	6
MLG17	1	USA	12	21	13	19	12	18	10	9	10	6	24	10	6	35	6	6
MLG18	1	USA	10	21	13	18	12	21	10	23	10	7	37	10	6	31	6	6
MLG19	1	USA	12	21	13	19	12	18	10	9	13	6	23	10	6	46	6	6
MLG20	1	USA	12	22	13	19	12	18	10	9	10	6	23	10	5	35	6	6
MLG21	2	USA	12	21	13	19	12	18	10	9	13	6	23	10	6	35	6	6
MLG22	1	USA	12	22	13	19	12	18	10	9	10	6	23	7	6	35	6	6
MLG23	1	ARG	12	22	13	18	13	21	10	18	10	8	15	12	6	23	11	6
MLG24	1	ARG	12	22	13	19	12	18	10	9	10	6	23	10	6	37	6	6
MLG25	1	ARG	12	22	13	18	13	21	10	18	10	8	19	12	6	30	11	6
MLG26	1	ARG	12	22	13	18	13	21	10	18	10	8	16	12	6	30	11	6
MLG27	1	USA	12	22	13	19	12	18	10	9	11	6	23	10	6	36	6	6
MLG28	1	USA	10	21	13	18	12	21	9	18	11	6	37	10	6	30	6	11
MLG29	1	USA	12	22	13	19	12	18	10	9	10	6	24	10	6	36	6	6
MLG30	1	USA	12	22	13	19	12	18	10	9	10	6	16	10	6	35	6	6
MLG31	1	USA	12	21	13	19	12	18	10	9	10	6	23	10	6	39	6	6
MLG32	1	USA	12	21	13	19	12	18	10	9	10	6	25	10	6	35	6	6
MLG33	1	USA	12	21	13	19	12	18	10	9	10	6	23	10	6	46	6	6
MLG34	2	ARG	12	11	13	18	13	21	10	18	10	8	15	12	6	30	11	6
MLG35	1	USA	12	22	13	18	13	21	10	18	11	6	15	12	6	30	11	6
MLG36	1	USA	12	23	13	18	13	21	10	18	12	6	15	12	6	30	11	6
MLG37	1	USA	12	23	13	19	12	18	10	9	10	6	23	10	6	35	6	6
**Number of alleles**			2	4	2	2	4	2	2	3	4	3	8	3	2	10	2	2

**Table 4:** Analysis of molecular variance (AMOVA) for *Fusarium virguliforme* populations from Argentina and the USA with or without clone-correction.

Source of Variation		Degree of Freedom	Variance Components	Percentage of Variation	F_ST_	*p*-Value
By strains	Among populations	1	53.195	17.20	0.17198	0.00098
	Within populations	88	256.109	82.80		
	Total	89	309.304			
By MLG	Among populations	1	27.187	37.11	0.37109	0.00000
	Within populations	36	95.734	62.89		
	Total	37	122.921	422.839		

**Table 5:** Genetic diversity in populations of *Fusarium virguliforme*.

Population	N ^a^	MLG ^b^	H ^c^
All	90	37	0.35
USA	56	27	0.33
Argentina	34	11	0.31
Cluster I	34	17	0.24
Cluster IA	5	4	0.30
Cluster IB	29	13	0.11
Cluster II	56	20	0.10

^a^ Number of strains; ^b^ Number of multilocus genotypes; ^c^ Nei’s gene diversity [28].

## Data Availability

The data presented in this study are available in the paper and Supplemental Documents.

## Supplementary Materials

The following supporting information can be downloaded at: https://www.mdpi.com/article/10.3390/jof9111109/s1. Table S1: Summary of microsatellite loci identified in *Fusarium virguliforme* strain Mont-1 by MISA. Table S2: Primers for the 29 informative microsatellite loci in *Fusarium virguliforme*. Table S3: ID matching of microsatellite loci identified in our study and those in a previous study [15]. Figure S1: PCR amplification of 29 informative microsatellite loci in four *Fusarium virguliforme* strains. Microsatellite ids are listed at the top. Lane number: M, 1kb DNA ladder; B, negative control; 1, Mont-1; 2, NRRL34551; 3, LL0009; and 4, Clinton-1B. Figure S2: Confirmation of strain identity as *Fusarium virguliforme* using species-specific PCR as previously described [21]. Figure S3: The number of multilocus genotypes (MLGs) detected in *Fusarium virguliforme* by randomly sampling of a subset of 16 microsatellite markers. Figure S4: Delta K graph for each K cluster.
