# Supplementary material for: Genome-Wide Informative Microsatellite Markers and Population Structure of Fusarium virguliforme from Argentina and the USA
Source: J Fungi (Basel). 2023 Nov 16;9(11):1109. doi: 10.3390/jof9111109 (PMC10672573; doi:10.3390/jof9111109)
Supplement: Supplementary file 1 [file jof-09-01109-s001.zip › Supplemental Figures.pdf]

Supplemental Figures:

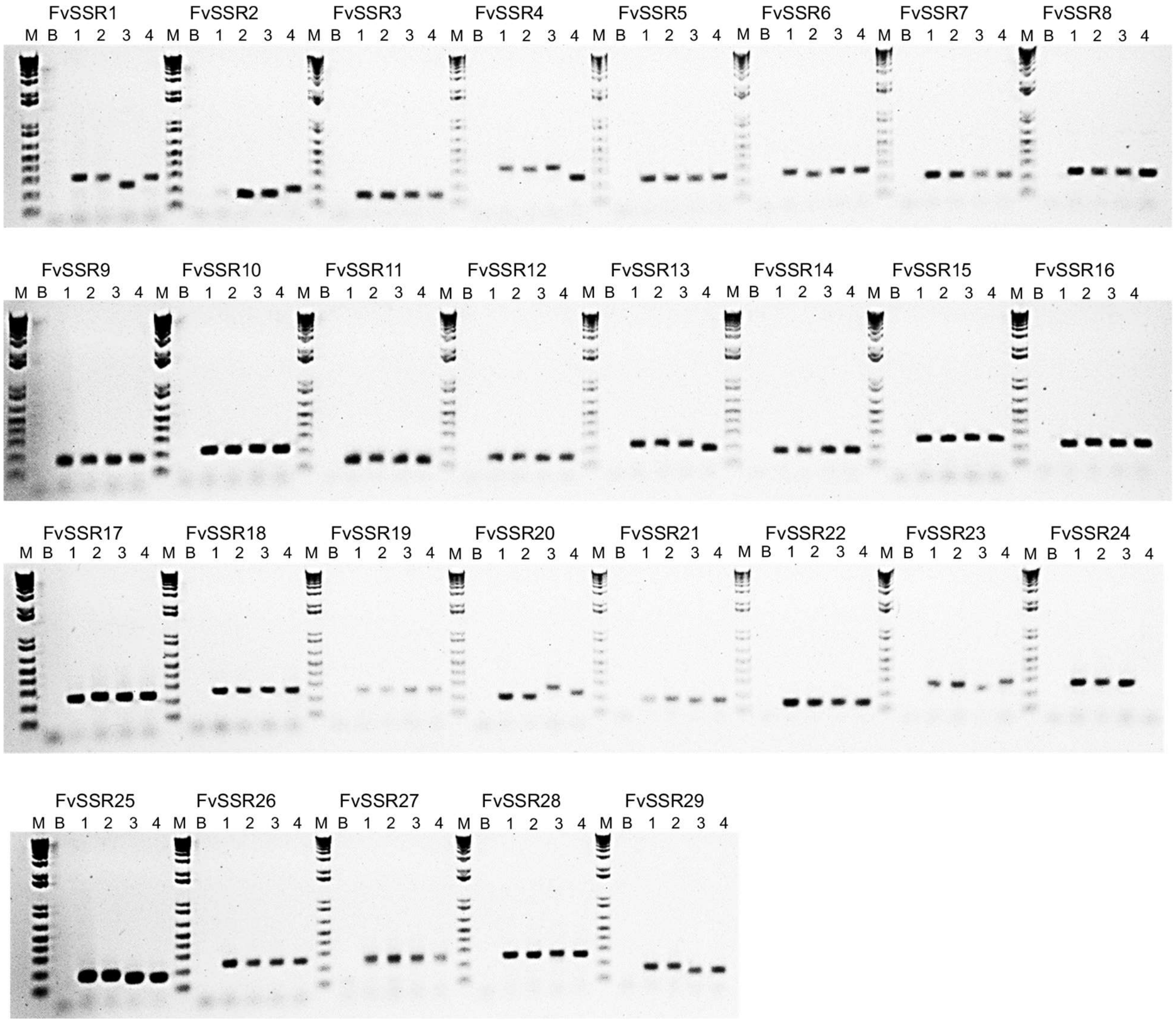

Figure S1. PCR amplification of 29 informative microsatellite loci in four *Fusarium virguliforme* strains. Microsatellite ids are listed at the top. Lane number: M, 1kb DNA ladder; B, negative control; 1, Mont-1; 2, NRRL34551; 3, LL0009; and 4, Clinton-1B.

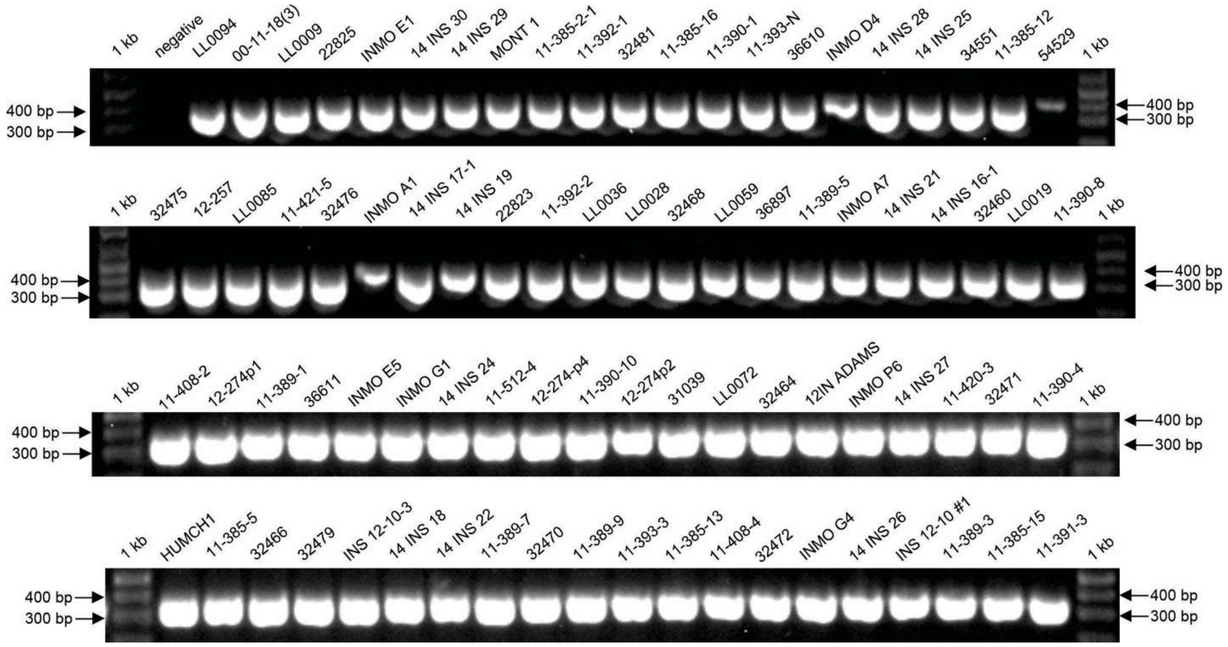

Figure S2. Confirmation of strain identity as *Fusarium virguliforme* using species-specific PCR as previously described [21].

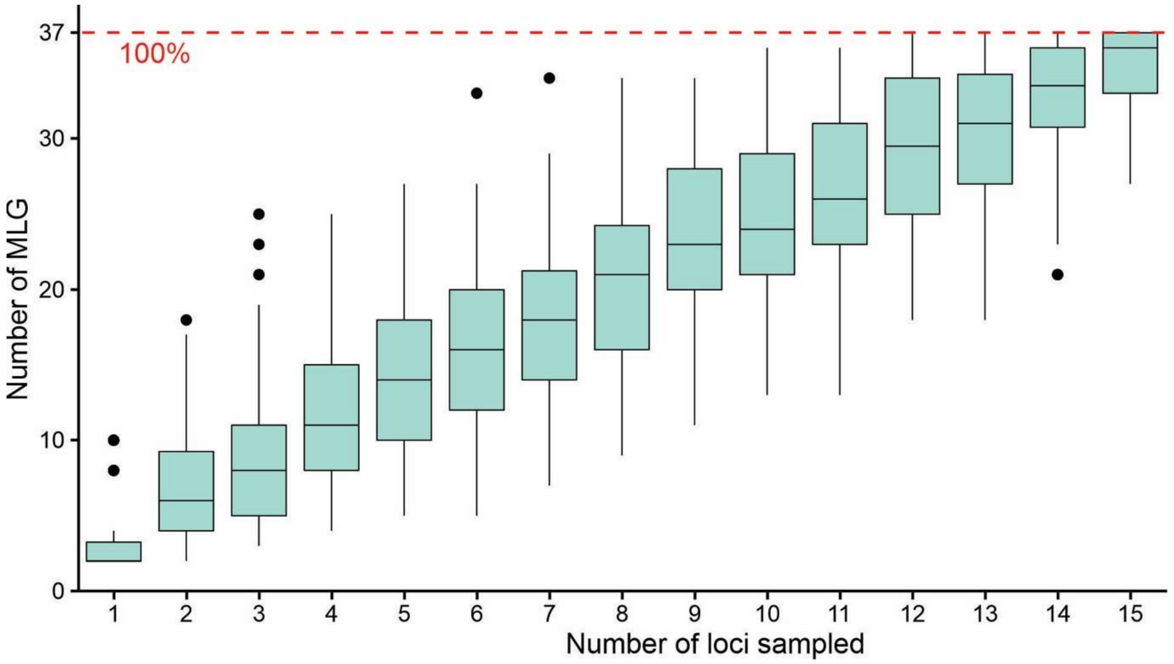

Figure S3. The number of multilocus genotypes (MLGs) detected in *Fusarium virguliforme* by randomly sampling of a subset of 16 microsatellite markers.

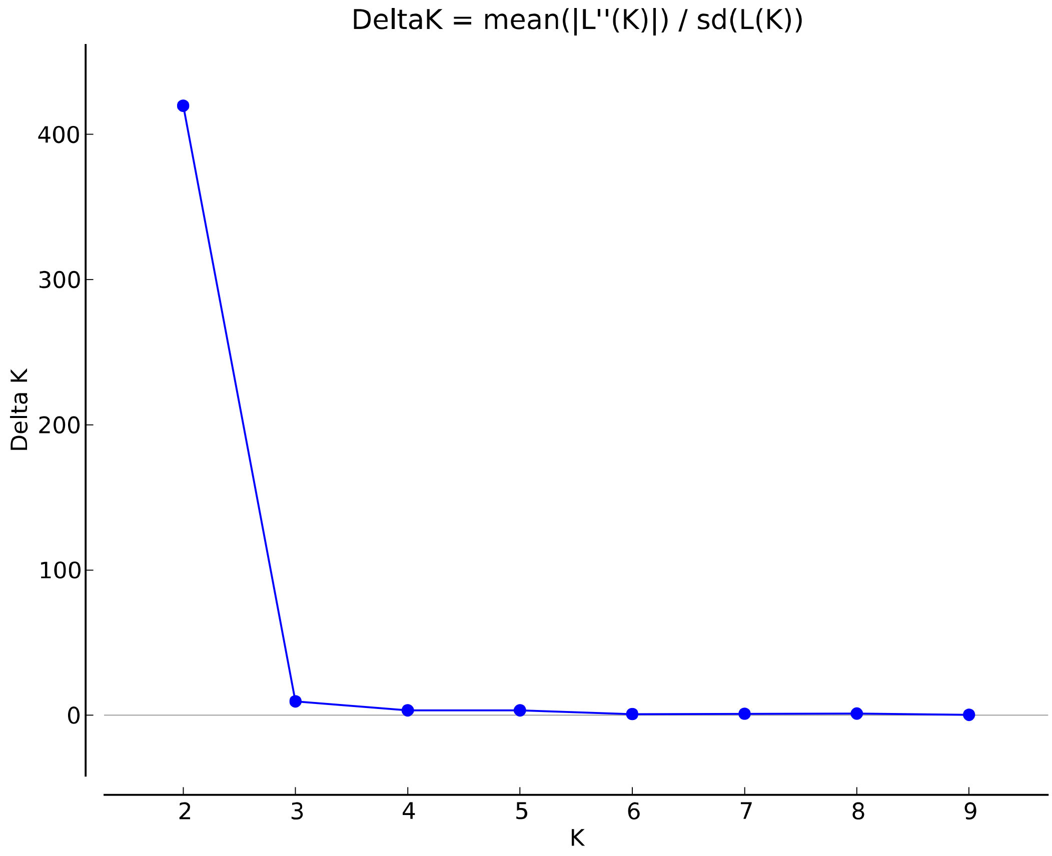

Figure S4. Delta K graph for each K cluster.
