# Supplementary material for: Genome-Wide Informative Microsatellite Markers and Population Structure of Fusarium virguliforme from Argentina and the USA
Source: J Fungi (Basel). 2023 Nov 16;9(11):1109. doi: 10.3390/jof9111109 (PMC10672573; doi:10.3390/jof9111109)
Supplement: Supplementary file 1 [file jof-09-01109-s001.zip › Tables S2-S3.pdf]

Table S2. Primers for the 29 informative microsatellite loci in *Fusarium virguliforme*.

Loci[a]	Primer name	Primer sequence[b]
SSR1	FvSSR1F	TGTAAAACGACGGCCAGTCGTCGTTGGCATTGTCCTTG
	FvSSR1R	GGCCGTATCCTAGGAGGTGA
SSR2	FvSSR2F	TGTAAAACGACGGCCAGTATTTCGACTTGCTCGTTGCG
	FvSSR2R	TCCATACAACAGCCACGGAC
SSR3	FvSSR3F	TGTAAAACGACGGCCAGTCTGGTTGTGTGTGCATCGTG
	FvSSR3R	GCCAGCCCGAATAAGCAAAG
SSR4	FvSSR4F	TGTAAAACGACGGCCAGTGAAGAGACTGGGGCCGTAAG
	FvSSR4R	GGGTCAACTTGGGGAAGGAG
SSR5*	FvSSR5F	TGTAAAACGACGGCCAGTCTGAGCTGTCTGTCTGTCGG
	FvSSR5R	CAGCTGCTGTCTGTCTGTCA
SSR6*	FvSSR6F	TGTAAAACGACGGCCAGTTGTGTGTTGGAGCTGAGGAC
	FvSSR6R	AAAGGATCGTCATCGTCCGG
SSR7*	FvSSR7F	TGTAAAACGACGGCCAGTGCTTGGAAACTGCAGCGAAT
	FvSSR7R	TCACCGACAATCGATTGCCT
SSR8	FvSSR8F	TGTAAAACGACGGCCAGTTTTCGGTATCCTTACGGCCC
	FvSSR8R	AGAAGAGGATGCCGATGCTG
SSR9*	FvSSR9F	TGTAAAACGACGGCCAGTGTGATGAGACAGGCCAAGCT
	FvSSR9R	TTGTTCCCTCTGACACTCGC
SSR10*	FvSSR10F	TGTAAAACGACGGCCAGTTGTTTCATGCCTTGCGTGTG
	FvSSR10R	GCTGGCCAGTCGGATACAAT
SSR11*	FvSSR11F	TGTAAAACGACGGCCAGTTATCATCCAGGGGCTGCAAC
	FvSSR11R	CAGGCGGTTTTGTTTTCGGT
SSR12*	FvSSR12F	TGTAAAACGACGGCCAGTGACAGGCTACAGGGGAACAC
	FvSSR12R	ACAGCCAACATCCAGACTCG
SSR13*	FvSSR13F	TGTAAAACGACGGCCAGTCGGGCGAGTCTTCTTCTCTC
	FvSSR13R	GACCCATGAAGGCGTTGGTA
SSR14	FvSSR14F	TGTAAAACGACGGCCAGTCATCTCTCCCCACACACCAC
	FvSSR14R	CTCTTACTCCTTGAGGCCGG
SSR15*	FvSSR15F	TGTAAAACGACGGCCAGTGCTTAGGAGAAGTGGCCCTC
	FvSSR15R	CGCAGAGATAACCGAACCCA
SSR16	FvSSR16F	TGTAAAACGACGGCCAGTCACGCTCACTCCGAGTTTCT
	FvSSR16R	GAGGCAAGAGCGAGCAAAAG
SSR17*	FvSSR17F	TGTAAAACGACGGCCAGTCCTCTCACCAGTGTAGCTGC
	FvSSR17R	ACTAGAACCGTTCGTCGTCG
SSR18	FvSSR18F	TGTAAAACGACGGCCAGTACCTCGTTGAGCAGTTCTGG
	FvSSR18R	CCCGTCGTTCTCATCGTGAT
SSR19	FvSSR19F	TGTAAAACGACGGCCAGTAGGACTCTCTACTAAAGAAAAAGGT
	FvSSR19R	TCATAGTCAAACGGGCGTCA
SSR20*	FvSSR20F	TGTAAAACGACGGCCAGTTTGACGCATGGGACCAATGT
	FvSSR20R	CCTCGGCAGAAATGACAGGT

SSR21*	FvSSR21F	TGTAAAACGACGGCCAGTACCTCACAACATCCACACACT
	FvSSR21R	GTGCGAGGTTTGCTTGAGTG
SSR22*	FvSSR22F	TGTAAAACGACGGCCAGTCTTGCGGCTCCTCCTTCTTA
	FvSSR22R	GGGATTCTCCAATGGCGGAT
SSR23*	FvSSR23F	TGTAAAACGACGGCCAGTTGGCTTCATCAACAGCTCCA
	FvSSR23R	GGGTCGTCTGGTGTTGAGAA
SSR24	FvSSR24F	TGTAAAACGACGGCCAGTTCTTTGGTGCCGAGGGAATC
	FvSSR24R	TCGAAGCAACCATTACGCCT
SSR25	FvSSR25F	TGTAAAACGACGGCCAGTGGTCCCAACCCCAAAGTAGG
	FvSSR25R	AAGGTGATGCGGGATGGATC
SSR26	FvSSR26F	TGTAAAACGACGGCCAGTCCTACAAAGGCTATACTGGGAAT
	FvSSR26R	TGGCACGGACGAAAATCAGA
SSR27	FvSSR27F	TGTAAAACGACGGCCAGTAGCCTAAATAGCTAGAGTAAAAGGA
	FvSSR27R	TGCTTACCGCTGAGATCCTT
SSR28*	FvSSR28F	TGTAAAACGACGGCCAGTGATTGGGCTGCTTTAAAAGACT
	FvSSR28R	AAAAGCTAGCACCATAACCCT
SSR29	FvSSR29F	TGTAAAACGACGGCCAGTGGAATAAAGAGGATTAGGGCCCT
	FvSSR29R	AGAACCCACTATATAGCCCGA
	M13	5'FAM-TGTAAAACGACGGCCAGT

[a] Loci labeled with * were used in the population study.

[b] M13 sequence was appended to the 5’ end of the forward primer.

Table S3: ID matching of microsatellite loci identified in our study and those in a previous study [15].

ID in this study	ID in Wang and Chilvers 2016
SSR1	48
SSR2	38
SSR4	43
SSR6	4
SSR13	93
SSR15	59
SSR20	80
SSR21	83
SSR23	10
